# *Spongionella* Secondary Metabolites, Promising Modulators of Immune Response through CD147 Receptor Modulation

**DOI:** 10.3389/fimmu.2016.00452

**Published:** 2016-10-24

**Authors:** Jon Andoni Sánchez, Amparo Alfonso, Ines Rodriguez, Eva Alonso, José Manuel Cifuentes, Roberto Bermudez, Mostafa E. Rateb, Marcel Jaspars, Wael E. Houssen, Rainer Ebel, Jioji Tabudravu, Luís M. Botana

**Affiliations:** ^1^Departamento de Farmacología, Facultad de Veterinaria, Universidad de Santiago de Compostela, Lugo, Spain; ^2^Departamento de Anatomía, Facultad de Veterinaria, Universidad de Santiago de Compostela, Lugo, Spain; ^3^Department of Chemistry, Marine Biodiscovery Centre, University of Aberdeen, Aberdeen, Scotland, UK; ^4^Pharmacognosy Department, Faculty of Pharmacy, Beni-Suef University, Beni-Suef, Egypt; ^5^Institute of Medical Sciences, University of Aberdeen, Aberdeen, Scotland, UK

**Keywords:** *Spongionella* sp., CD147, cyclophilin A, inflammation, chemotaxis

## Abstract

The modulation of the immune system can have multiple applications such as cancer treatment, and a wide type of processes involving inflammation where the potent chemotactic agent cyclophilin A (Cyp A) is implicated. The Porifera phylum, in which *Spongionella* is encompassed, is the main producer of marine bioactive compounds. Four secondary metabolites obtained from *Spongionella* (Gracilin H, A, L, and Tetrahydroaplysulphurin-1) were described to hit Cyp A and to block the release of inflammation mediators. Based on these results, some role of *Spongionella* compounds on other steps of the signaling pathway mediated by this chemotactic agent can be hypothesized. In the present paper, we studied the effect of these four compounds on the surface membrane CD147 receptor expression, on the extracellular levels of Cyp A and on the ability to migrate of concanavalin (Con A)-activated T lymphocytes. Similar to a well-known immunosuppressive agent cyclosporine A (CsA), Gracilin H, A, L, and tetrahydroaplysulphurin-1 were able to reduce the CD147 membrane expression and to block the release of Cyp A to the medium. Besides, by using Cyp A as chemotactic agent, T cell migration was inhibited when cells were previously incubated with Gracilin A and Gracilin L. These positive results lead us to test the *in vivo* effect of Gracilin H and L in a mouse ear delayed hypersensitive reaction. Thus, both compounds efficiently reduce the ear swelling as well as the inflammatory cell infiltration. These results provide more evidences for their potential therapeutic application in immune-related diseases of *Spongionella* compounds.

## Introduction

Cyclophilins (Cyps) are proteins ubiquitously distributed intracellularly in all prokaryotic and eukaryotic cells. The description of cyclophilin (Cyp) inhibitors began in 1970 when cyclosporine A (CsA) was first isolated from the fungus *Tolypocladium inflatum*, and then its inhibitory effect over T-cell reactivity was observed (1). Nevertheless, it was not until 1984 when the first Cyp, specifically cyclophilin A (Cyp A), was purified from bovine thymocytes, and it was confirmed as the primary intracellular receptor of the immunosuppressive drug CsA (2). This protein family has been shown to possess peptidyl-prolyl *cis-trans* isomerase (PPIase) activity and plays an important role intracellularly in protein folding and trafficking (3). Although it was initially believed that Cyps exist only as intracellular proteins, nowadays it is known that some members of this family can be secreted to the extracellular media where they play different functions as mediators of intra and intercellular communication. The first Cyp discovered in extracellular fluids was Cyp B, specifically in milk and plasma (4). Shortly after, it was discovered that Cyp A is secreted to extracellular media in response to inflammatory stimuli (5).

Leukocyte trafficking and recruitment is a critical component in host immune surveillance and inflammation-mediated pathology. The main regulators of leukocyte trafficking are chemokines, a family of chemoattracting cytokines that control cell migration and adhesion (6). However, other factors like extracellular Cyp A have been described by different reports as potent chemotactic response inducers in human monocytes, neutrophils, eosinophils, and T cells either by *in vitro* or *in vivo* assays (7). Secreted Cyp A can initiate signaling response in target cells. In this sense, the binding protein receptor that transduces Cyp A-activated signal into target cells is the CD147 receptor also called extracellular matrix metalloproteinase inducer (EMMPRIN) or Basigin (8). Moreover, Cyp A is not only the ligand for CD147 receptor but also is responsible for the translocation of CD147 to the membrane surface (9).

Although CD147 receptor is expressed in leukocytes and its implication has been investigated in deep in inflammatory response, it is ubiquitously expressed in almost all studied cell types, such as hematopoietic, epithelial, endothelial, or tumor cells in different levels (10). In the case of tumor cells, CD147 has multiple functions related with metastasis or angiogenesis, while in normal cells it is related with a wide range of processes as immune/inflammatory response (11). In an inflammatory process, the expression of CD147 is upregulated (12–14). The expression of this receptor can also be induced *in vitro* whether T cell is activated through its TCR by mitogens, such as lectins (15).

Sponges are aquatic organisms able to adapt to different environments (16). A wide type of bioactive compounds had been isolated from these invertebrate animals (17). Anti-inflammatory, immunosuppressive, anticoagulant, antibacterial, antifungal, or anticancer effects had been associated to some molecules obtained from sponges (18). In this sense, bisnorditerpene Gracilin H, norditerpene Gracilin A, 12-hydroxy derivative Gracilin L, and the diterpenoid 3′-norspongionlactone tetrahydroaplysulphurin-1 were isolated from the marine sponge *Spongionella gracillis*. These compounds have shown interesting antioxidant effects and the inhibition of Alzheimer hallmarks (19, 20). In addition, Gracilin H, A, L, and Tetrahydroaplysulphurin-1 were able to form complexes with intracellular Cyps (21). In the case of Cyp A, *Spongionella* compounds showed a *K*_D_ in the micromolar range, similar to CsA, a well-known Cyp A binder, and in the same way, they regulated transcriptional factors and the production of interleukin 2 (IL-2) (21, 22). Thus, these compounds could mimic other CsA effects related to the immune response. In this context, the aim of this work was to deeply study the modulatory effect of *Spongionella-*isolated compounds on Cyp A functions, different than calcineurin blockade, such as CD147 receptor expression and chemotaxis.

## Materials and Methods

### Chemicals

Human Cyp A Elisa kit was purchased from Novateinbio (Woburn, MA, USA); FITC Anti-Human CD147 (Ref-14781-100T) and Isotype control IgG1 FITC (Ref-ICIGG1F-100T) was from Immunostep (Salamanca, Spain). QCM™ Chemotaxis 5 μm 96-Well Cell Migration Assays was from Millipore (Germany). Percoll was obtained from Pharmacia (Uppsala, Sweden). Plastic tissue-culture dishes were from Falcon (Madrid, Spain). RPMI and fetal calf serum (FBS) were bought from Gibco (Glasgow, UK). Methanol (HPLC grade) was supplied by Merck (Barcelona, Spain). The human Pan T cell Isolation Kit (Ref-130-096-535) and the monoclonal antibody to human CD3, clone BW264/56, FITC (Ref-130-098-162) were purchased from Miltenyi Biotec (Germany). Human IL-2 ELISA kit was obtained from Invitrogen (Spain). Active human Cyp A full-length protein was from Abcam (UK). Paraformaldehide (PFA) was from Aname (Madrid, Spain). CsA (purity ≥98.5%), 1-Fluoro-2, 4-dinitrobenzene (DNFB), poly-l-lisine, α-chymotrypsin from bovine pancreas, *N*-succinyl–Ala–Ala–Pro–Phe–*p*-nitroanilide, and the rest of chemicals and reagents were obtained from Sigma-Aldrich (Madrid, Spain). The composition of saline solution (PBS) used for human T lymphocytes purification was (in millimolar) 137 ClNa, 8.2 Na_2_HPO_4_, 1.5 KH_2_PO_4_, 3.2 KCl, and 2 EDTA. The composition of Umbreit saline solution was (in millimolar) NaCl 119, MgSO_4_ 1.2, NaH_2_PO_4_ 1.2, NaHCO_3_ 22.85, KCl 5.94, and CaCl_2_ 1. Glucose 1 g L^−1^ was added to the medium. The pH was equilibrated between 7.2 and 7.3. Stock solutions of drugs were done in dimethyl sulfoxide (DMSO).

Acetonitrile and methanol were supplied by Panreac (Barcelona, Spain). All solvents employed in this work were of HPLC or analytical grade, and the water was distilled and passed through a water purification system (Milli-Q, Millipore, Spain). Formic acid was purchased from Merck (Darmstadt, Germany). Ammonium acetate was from Fluka (Sigma-Aldrich, Spain).

### Human T Lymphocytes Isolation

Peripheral lymphocytes were isolated from human fresh heparinized blood from healthy volunteers as previously described (23). The blood was diluted in the same proportion with PBS plus EDTA 2 mM previously equilibrated at room temperature. Also, 4 mL of diluted blood were carefully placed over 3 mL of isotonic percoll (57.5%) previously disposed in 10-mL tubes, avoiding the mixture of phases. The tubes were centrifuged at 3000 rpm, 25 min at room temperature. After centrifugation, different phases were obtained, and only the fraction containing the population of lymphocytes was collected and washed three times with PBS–EDTA to remove percoll at 1500 rpm, 10 min, and room temperature. Lymphocyte purity was always higher than 80%. T lymphocytes were purified from this population with a Pan T cell Isolation Kit. This is an indirect magnetic labeling system for the isolation of untouched T cells. T cell purity was always higher than 95%. Assessment of cell purity was performed by flow cytometry by using a monoclonal antibody to human CD3 labeled with FITC. Viability (>95%) was determined by trypan blue exclusion. Pure T cells were maintained in RPMI 1640 plus 10% FBS and plated in 24 plastic tissue-culture dishes in humidified 5% CO_2_ and 95% air atmosphere at 37°C.

### CD147 Receptor Expression in T Lymphocytes

For CD147 measurements, T cells were pretreated with *Spongionella* compounds or CsA 2 h before Con A (50 μg mL^−1^) stimulation for 48 h. After this, cells were centrifuged at 2000 rpm, 5 min, and 4°C to remove RPMI 1640 culture medium. Next, cells were washed twice with PBS–BSA 2% (2000 rpm, 5 min, 4°C), and a final concentration of 1 × 10^6^ cells in 50 μL were incubated with 20 μL of FITC Anti-Human CD147 for 90 min in darkness and at 4°C in a automatic shaker. After this time, T cells were washed twice with PBS to remove the dye (2000 rpm, 5 min, 4°C) and resuspended in a final volume of 60 μL of PBS with 1% paraformaldehyde and kept on ice until use. Cells were attached onto 22-mm glass coverslips treated with poly-l-lysine for 10 min. FITC excitation and emission are 494/520 nm, respectively. Lasers used for excitation and emission were 476 and 510 with the confocal laser-scanning microscope Nikon D-Eclipse C1. The images were collected using the 40× immersion objective (Nikon).

For flow cytometry assays, cells were resuspended in a final volume of 2 × 10^6^ cells in 100 μL of PBS with 1% paraformaldehyde and kept on ice until use and analyzed by flow cytometry technique. The IDEAS ImageStream Analysis software was used to analyze the data obtained from the measurements of the fluorescence intensity.

### Cyp A Release Measurements

For Cyp A measurements, 2 × 10^6^ cells in a volume of 200 μL were seeded in a 96-well plate. Human T cells were pretreated with *Spongionella* compounds or CsA for 2 h. Then, cells were stimulated with Con A at 50 μg mL^−1^ for 48 h to induce Cyp A release to the culture medium. After the incubation time, cells were centrifuged, and the cell culture was collected and assayed immediately by using Human Cyclophilin A Elisa kit. The amount of Cyp A present in the culture medium was measured in a spectrophotometer at the wavelength of 450 nm.

### Chemotaxis Assays

The chemotaxis assays were conducted using purified human T lymphocytes. The chemotactic activity was assessed using QCM™ Chemotaxis 5 μm 96-Well Cell Migration Assays (Millipore) composed by two compartments separated by a 5-μm membrane. Treated cells (200,000 per condition) were located on the top of the insert membrane in RPMI 1640 culture medium in the absence of FBS for 24 h at 37°C and 5%CO_2_. During this time, cells can migrate to the feed chamber, located under the insert membrane, where the medium contains the chemoattractant human recombinant Cyp A (50 ng mL^−1^). After 24 h, the migratory cells were collected in 150 μL of medium that contains the feed chamber plus migratory cells on the bottom of the insert membrane (cells that cross the membrane but stay adhered to the bottom part) that are dissociated from the membrane after 30 min incubation with Cell Detachment Buffer. A volume of 75 μL of dissociated cells plus 75 μL of cells from the migration feeder are mixed. These cells are subsequently incubated for 15 min at room temperature with Lysed buffer that contains the CyQuant GR dye. After this time, the fluorescence of lysed T cells was determined in a microplate reader at 480 nm excitation and 520 nm emission in a spectrophotometer plate reader.

### Pharmacokinetic Assays

Swiss mice colonies were kept in the facilities of the animal care centre of Universidad de Santiago de Compostela (Spain). All animal protocols were carried out following the principles approved by the Institutional Animal Care Committee of the Universidad de Santiago de Compostela. For the experiments, three animals per group were used. The animals were fed *ad libitum* and maintained under controlled conditions with a temperature of 24°C, humidity 40–80%, and 12-h light and dark cycle. All procedures were in strict accordance with the Guide for the Care and Use of Laboratory Animals. All specimens were about 12 weeks old and weighed 20–25 g.

Mice were intraperitoneally (IP) injected with CsA or *Spongionella* compounds at 0.4 mg kg^−1^ and were housed in a metabolic cage where the urine and feces were collected at 1 and 24 h. Animals were sacrificed and blood, urine, feces samples, and brain were collected.

### Sample Extraction

Whole blood samples were collected in tubes, stored at −80°C until analysis, and thawed to room temperature before use. Tissue samples (brain) were weighed and homogenized in 1 mL of phosphate buffered saline 10 mM (PBS) using a manual homogenizer and centrifuged at 10,000 × *g* for 10 min. The supernatant was separated from the pellet and frozen at −80°C until analysis and thawed to room temperature before use. In the case of feces, 1 mL of PBS was added to the samples and mixed for 2 min to homogenate with the PBS. After these, feces were centrifuged at 10,000 × *g*, and the supernatant was stored at −80°C. In the case of urine, per 100 μL of sample, 20 μL of methanol was added and also stored at −80°C. Afterward, all samples were processed with the same method. To 100 μL of mixture, 40 μL of methanol were added and vortex-mixed for 30 s. Then, 10 μL of 10% trichloroacetic acid aqueous solution were added and vortex-mixed for 30 s. Later, 50 μL of acetonitrile were added to the mixture and vortex-mixed for 1 min. Samples were centrifuged at 14,500 × *g* for 15 min, and the supernatant was filtered with 0.22-μm filter and injected (5 μL) in the LC column.

The recuperation rate of the method was calculated as 83% recovery for CsA and 84% recovery for Gracilin H and L, except in blood with a 60% recovery.

### LC–MS/MS Analysis

For CsA analysis, a 1290 Infinity ultra-high-performance liquid chromatography (UHPLC) system coupled to a 6460 Triple Quadrupole mass spectrometer (both Agilent Technologies, Waldbronn, Germany) was used. Chromatographic separation was performed at 50°C, the injection volume was 5 μL, and flow rate of 0.4 mL min^−1^ using a column AQUITY UPLC BEH C18 (2.1 mm × 100 mm, 1.7 μm, Waters, Spain). The nitrogen generator is a Nitrocraft NCLC/MS from Air Liquid (Spain). The mobile phase A was water with 0.1% formic acid and 2 mM ammonium acetate, and the mobile phase B was methanol. Chromatographic separation was performed by gradient elution starting with 80% B for 1.2 min, then increasing to 95% B for 4 min, this condition was held for 5 min, and reduced afterward to 80% B over 0.2 min. This proportion was maintained for 1.8 min until the next injection to equilibrate the system. Source conditions were optimized to achieve the best sensitivity for CsA. A drying gas temperature of 250°C and a flow gas of 5 L min^−1^, sheath gas temperature of 250°C, a sheath gas flow of 11 L min^−1^, and nebulizer gas pressure of 30 psi were used. The capillary voltage was set to 4500 V with a nozzle voltage of 500 V. The fragmentor was 165 for *m*/*z* 1219.87 and 140 for *m*/*z* 601.93, and the cell accelerator voltage was 4 V. The collision energy, optimized using MassHunter Optimizer software, was 12 V for *m*/*z* 1219.87 and 32 and 60 V for *m*/*z* 601.93. Analysis was carried out using electrospray ionization (ESI) and multiple reactions monitoring (MRM) in positive mode (Table 1). Three product ions were analyzed, one for quantification and another for confirmation. The transitions employed were *m*/*z* 1219.87 > 1202.8, *m*/*z* 601.93 > 156.1/100.1. For CsA standard, an eight-points calibration curve among the range 3.91–500 ng mL^−1^ was done. The limit of detection (LOD) was 1.5 ng mL^−1^, and the limit of quantification (LOQ) was 3.91 ng mL^−1^.

**Table 1:** **Quantification of CsA, Gracilin H, and Gracilin L obtained by UPLC-MS method in four matrixes of blood, brain, urine, and feces at 1 and 24 h after IP injection**.

	Blood (ng/100 μL)		Brain (ng/brain)		Urine (ng/mL)		Feces (ng/g)	
	1 h	24 h	1 h	24 h	1 h	24 h	1 h	24 h
CsA	419.87 ± 45.4	9.3 ± 0.3	36.1 ± 6.8	9.2 ± 0.3	441 ± 112	62.3 ± 19.7	80.47 ± 27.6	14.5 ± 2.8
Gracilin H	nd	nd	nd	nd	6 ± 3.3	3.5 ± 1.5	38.1 ± 19.4	<LOQ
Gracilin L	nd	nd	nd	nd	52.23 ± 7.3	13.29 ± 7.3	nd	nd

*LOQ, limit of quantification; nd, not detected*.

### LC–PDA–IT–TOF Analysis

For Gracilins analysis de UPLC-IT-TOF was used. The UPLC system, from Shimadzu (Kyoto, Japan) consists of two pumps (LC-30AD), autoinjector (SIL-10AC) with refrigerated rack, degasser (DGU-20A), column oven (CTO-10AS), and a system controller (SCL-10Avp). The system is coupled to an IT–TOF–MS system with an ESI interface (Shimadzu, Kyoto, Japan) and Photodiode Array UV-Vis detector (SPD-M20A). The nitrogen generator is a Nitrocraft NCLC/MS from Air Liquid (Spain). The molecules were separated using a column AQUITY UPLC BEH C18 (2.1 mm× 100 mm, 1.7 μm, Waters, Spain) at 35°C. Mobile phase A was 100% water with 0.1% formic acid. Mobile phase B was acetonitrile 100%, containing 0.1% formic acid. The gradient run started at 50% B (0–7 min), raised to 100% B (7–8 min), held to 100% B (8–9 min), returned to 50% B (9–9.5 min), and held to 50% B (9.5–12 min). The samples in the autosampler were cooled to 4°C, and injection volume was 5 μL. The MS method was operated in positive mode with the following ESI source conditions: nebulizing gas flow, 1.5 L min^−1^, heat block temperature and CDL temperature, 200°C, and detector voltage, 1.65 kV. The molecules were analyzed using an ion accumulation time of 50 ms. The cell of PDA detector maintained at 30°C. The range of wavelength of the PDA detector was set at 190–600 nm because of the deuterium (D_2_) lamp used.

For Gracilin H and Gracilin L standard, an eight-points calibration curve among the range 3.91–500 ng mL^−1^ was done. The LOD was 1.5 ng mL^−1^, and the LOQ was 3.91 ng mL^−1^.

### DNFB-Induced Delayed-Type Hypersensitivity Reaction

The DNFB-induced delayed-type hypersensitivity (DTH) reaction assay was performed as previously described (24). On days 0 and 1, mice were sensitized by skin painting on the abdomen with 25 μL of 0.5% DNFB dissolved in acetone–olive oil (4:1). Vehicle, CsA (used as the positive reference substance), or *Spongionella* compounds were IP administered to each group (*n* = 3) on five consecutive days (days 2–6) at 0.4 mg kg^−1^. On day 6, mice were challenged by the application of 20 μL of 0.2% DNFB on inner and outer surfaces of the right ear, while the left ear was used as control of normal tissue. After 24 h, the difference in thickness between the left and the right constant area of the ear was measured with a caliper. The increment rate of ear swelling was expressed as follows: % of increment = [(thickness of the right ear − thickness of the left ear)/thickness of the left ear] × 100. Mice were sacrificed, and the right ear tissue was fixed by immersion in 10% buffered formalin solution. Then, samples were embedded in paraffin according to standard laboratory procedures, and (3 μm) sections were mounted onto silanized slides and dried overnight at 37°C. Tissue sections were routinely stained with Mayer’s hematoxylin and eosin (H-E) dyes following standard procedures for routine histological evaluations.

### Statistical Analysis

All experiments were carried out by duplicate a minimum of three times. Results were analyzed by using one-way analysis of variance ANOVA with Dunnett’s *post hoc* analysis. Also, *p* values <0.05 were considered statistically significant. All results were expressed as the mean ± SEM of three or more experiments.

## Results

In a previous work, we demonstrated that Gracilin H, A, L, and Tetrahydroaplysulphurin-1 were able to form a complex with intracellular Cyp A. All *Spongionella* compounds showed a *K*_D_ in the micromolar range, similar to CsA, a well-known Cyp A binder (22). Thus, considering these previous results we studied in which extent *Spongionella* compounds modulate other functions such as extracellular Cyp A and CD147 receptor functions.

**Figure 1:**
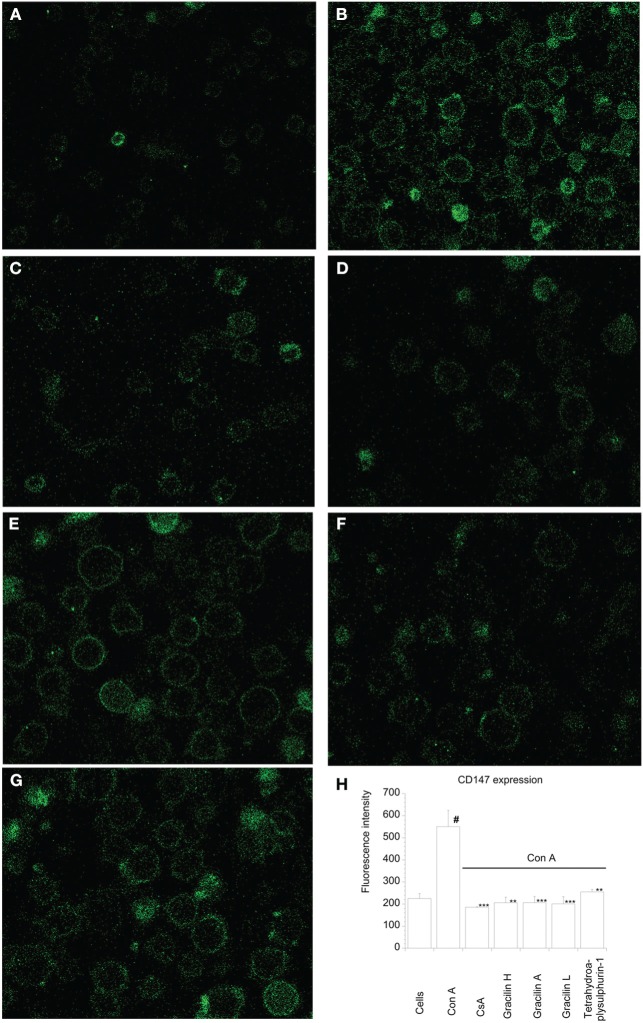
**Effect of CsA or *Spongionella* compounds on CD147 receptor levels in human T lymphocyte membrane stimulated with Con A**. Confocal microscopy representative images from the experiments that correspond to **(A)** untreated control cells, **(B)** human T lymphocytes stimulated with Con A (50 μg mL^−1^) for 48 h, **(C)** human T lymphocytes pretreated for 2 h with CsA (0.2 μM) before Con A stimulation for 48 h, or **(D)** Gracilin H (1 μM), **(E)** Gracilin A (1 μM), **(F)** Gracilin L (1 μM), or **(G)** Tetrahydroaplysulphurin-1 (1 μM), **(H)** fluorescence intensity of human T lymphocytes pretreated for 2 h with CsA (0.2 μM), Gracilin H (1 μM), Gracilin A (1 μM), Gracilin L (1 μM), or Tretrahydroaplysulphurin-1 (1 μM) and then with Con A (50 μg mL^−1^) for 48 h. All values are presented as the mean fluorescence intensity and are compared to that of T lymphocytes treated with Con A 50 μg mL^−1^ alone by ANOVA statistical analysis followed by *post hoc* Dunnet’s *t*-test. *p < 0.05, **p < 0.01, ***p < 0.001, or between control cells and Con A-treated cells ^#^*p* < 0.05. Data are the mean ± SEM of three independent experiments carried out by duplicate.

**Figure 2:**
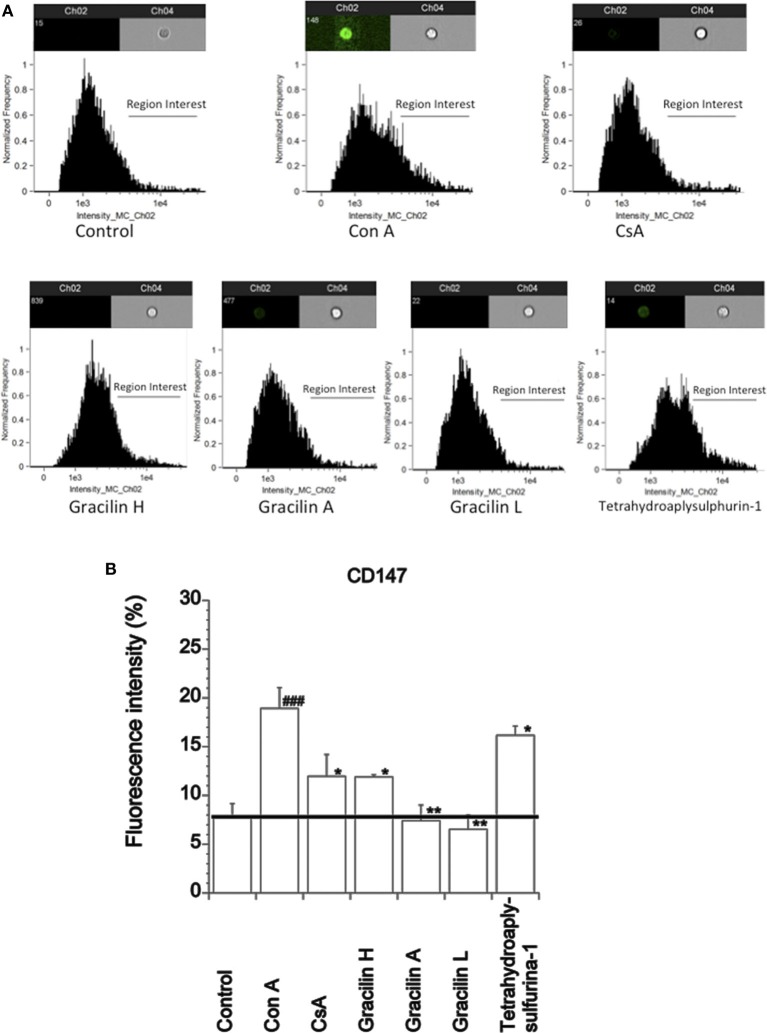
**Effect of CsA or *Spongionella* compounds on plasma membrane levels of CD147 receptor in human T lymphocytes stimulated with Con A**. **(A)** Representative cellular images and histograms of the CD147 receptor expression of untreated human T lymphocyte control cells, Con A (50 μg mL^−1^) for 48 h treated cells and human T lymphocytes pretreated for 2 h with CsA (0.2 μM) or *Spongionella* compounds (1 μM) before Con A stimulation for 48 h. Brightfield images are represented in channel 4, and FITC intensity images are shown in channel 2. **(B)** Percentage of cells with CD147 expression in plasma membrane after Con A or CsA (0.2 μM), Gracilin H, A, L, and tetrahydroaplysulfurin-1 pretreated for 2 h with at 1 μM before Con A stimulation for 48 h. All values are presented as the percentage of fluorescence intensity and are compared to that of T lymphocytes treated with Con A 50 μg mL^−1^ alone by ANOVA statistical analysis followed by *post hoc* Dunnet’s *t*-test. *p < 0.05, **p < 0.01, or between control cells and Con A-treated cells ^###^*p* < 0.001. Data are the mean ± SEM of three independent experiments (10,000 cells were analyzed in each experiment).

In normal tissues, T lymphocytes ubiquitously express CD147 receptor. Nevertheless, under pathological situations, T cells can overexpress this receptor on its surface (25). In addition, the translocation and expression of CD147 receptor to the membrane is mediated by Cyp A, and it is greatly reduced after CsA treatment (25). Therefore, we first checked the effect of *Spongionella* compounds over CD147 expression. The increase of CD147 expression can also be *in vitro* induced after T lymphocytes activation (15). In this way, we first measured the baseline expression of CD147 in non-activated versus activated T lymphocytes by confocal microscopy with Anti-Human CD147 FITC-labeled. As Figure 1H shows, while in resting cells the average of fluorescence intensity corresponding to CD147 expression was 225 ± 22.4 fluorescence units, in T cells, after 48 h stimulation in the presence of Con A, the fluorescence was significantly increased to 550.79 ± 73.1 fluorescence units (*p* < 0.05), that is 2.44 times. Therefore, *in vitro* Con A-activated T cells expressed higher levels of CD147 than resting cells. A representative microscopy image shows the difference in fluorescence intensity in activated (brightly fluorescent) (Figure 1B) versus non-activated T cells (Figure 1A). When T cells were pretreated with CsA (used as positive control) 2 h before Con A stimulation (Figures 1C,H), the fluorescence intensity units decreased to 185.7 ± 1.98 (*p* < 0.001), similar levels than resting cells. Straightaway, the effect of *Spongionella* compounds pretreatment before Con A activation was also tested. With similar potency than CsA, all compounds were able to reduce the fluorescence intensity to control cell levels; Gracilin H, A, L, and Tetrahydroaplysulphurin-1 decreased the fluorescence intensity unit values to 206.23 ± 25.2 (*p* < 0.01), 206.24 ± 28.3 (*p* < 0.001), 201.34 ± 32.2 8 (*p* < 0.001), and 255.27 ± 11.6 (*p* < 0.01), respectively (Figures 1D–H). Therefore, *Spongionella* compounds were able to efficiently reduce the expression of CD147. These results were confirmed by flow cytometry (Figure 2A). The fluorescence of Anti-Human CD147 labeled with FITC was measured in channel 2, thus the intensity in this channel is proportional of CD147 receptor expression. As Figure 2B shows, 7.84 ± 1.31% of control cells constitutively express CD147 receptor, while in Con A-treated cells this population was increased to 18.94 ± 2.1% (*p* < 0.001). When cells were treated with CsA or *Spongionella* compounds before activation, the percentage of CD147 positive cells significantly decreased to 11.9 ± 2.22% (*p* < 0.05) in the case of CsA and to 7.41 ± 1.6% (*p* < 0.01) and 6.54 ± 1.46 (*p* < 0.01) in the case of Gracilin A and L, respectively, while Tetrahydroaplysulfurin-1 incubation produced the lower reduction of CD147 positive cells, 16.17 ± 0.95% (*p* < 0.05). Therefore, *Spongionella* compounds significantly reduce the presence of CD147 receptor on the cellular membrane of T cells.

**Figure 3:**
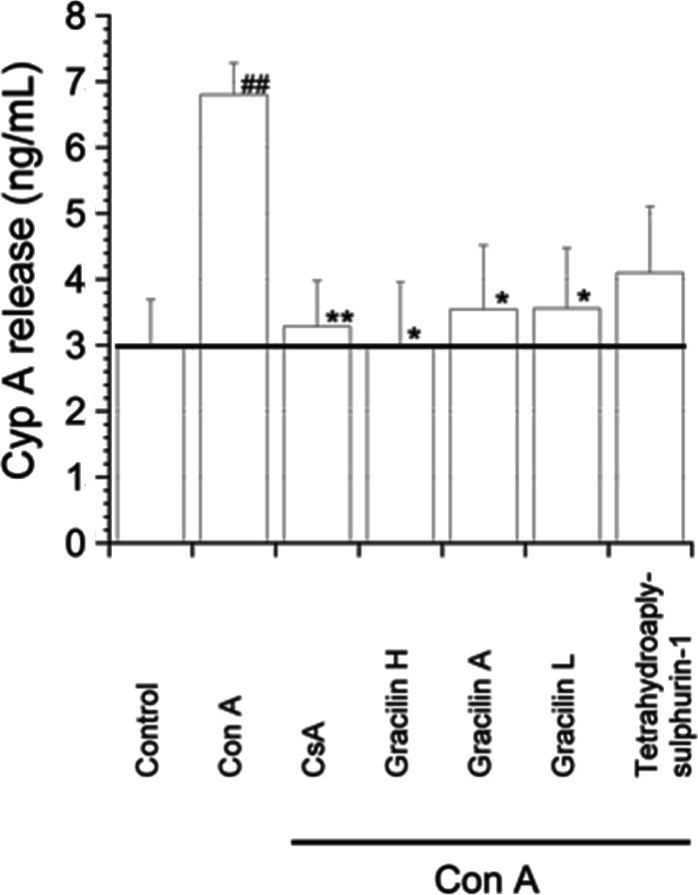
**Effect of CsA or *Spongionella* compounds on Cyp A release in human T lymphocytes stimulated with Con A**. Human T lymphocytes were pretreated for 2 h with CsA (0.2 μM), Gracilin H (1 μM), Gracilin A (1 μM), Gracilin L (1 μM), or Tetrahydroaplysulphurin-1 (1 μM), and then with Con A (50 μg mL^−1^) for 48 h. All values are shown in percentage with respect to Con A-treated cells and compared to cells treated with CsA or *Spongionella* compounds by ANOVA statistical analysis followed by *post hoc* Dunnet’s *t*-test. *p < 0.05, **p < 0.01 or Con A-treated cells versus control cells ^##^*p* < 0.01. Mean ± SEM of three independent experiments.

Cyclophilin A acts as a strong chemotactic substance. In the case of inflammatory cells, under pathological situations, the cells can secrete this protein to the medium (7). Once in the extracellular medium, Cyp A acts recruiting and attracting to the damage tissues the inflammatory cells where they develop their functions. Thus, T cells are also attracted, and CD147 receptor acts as Cyp A binder (26). We first checked the differences in secretion of Cyp A to the extracellular medium between activated and resting T cells. As Figure 3 shows, there was a significant increase in the Cyp A release from activated T cells to the medium, 2.97 ± 0.72 ng mL^−1^ from control cells and 6.8 ± 0.47 ng mL^−1^ (*p* < 0.01) from Con A-activated T lymphocytes. When the cells were 2 h pretreated with CsA or *Spongionella* compounds, the Cyp A release was decreased almost to control conditions, although in the case of Tetrahydroaplysulphurin-1, the decrease was not significant.

**Figure 4:**
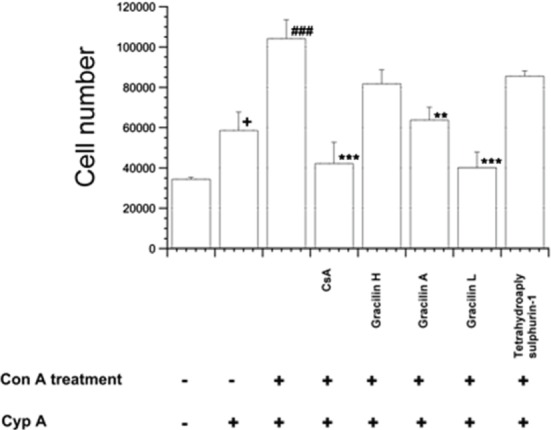
**Quantification of human T lymphocytes migration**. Human T lymphocytes were pretreated for 2 h with CsA (0.2 μM), Gracilin H (1 μM), Gracilin A (1 μM), Gracilin L (1 μM), or Tetrahydroaplysulphurin-1 (1 μM), and then with Con A (50 μg mL^−1^) for 48 h. After the treatment, T cells were placed in the migration chamber for 24 h in the presence of the chemotactic agent Cyp A (50 ng mL^−1^). Control of non-activated T cells in the presence or absence of Cyp A is also included. All values are shown as the difference between Con A migratory cell number versus Con A and CsA or *Spongionella* compounds treated cells in the presence of Cyp A by ANOVA statistical analysis followed by *post hoc* Dunnet’s *t*-test. *p < 0.05, **p < 0.01, ***p < 0.001, or Con A-treated cells in the presence of Cyp A versus control cells ^###^*p* < 0.001 or Con A-treated cells versus non-stimulated cells in the presence of Cyp A ^+^*p* < 0.05. Mean ± SEM of three independent experiments.

The next question was the correlation between CD147 expression and the Cyp A-mediated immune response. Thus, the capacity of human Cyp A to induce migration in populations of T lymphocytes was checked. In these studies, human T lymphocytes were stimulated *in vitro* with Con A for 48 h to generate populations containing high CD147 expression. After activation, T cells were placed in the upper part of a migration chamber with human Cyp A (50 ng mL^−1^) in the bottom part. As Figure 4 shows, from 200,000 cells, 34,428 ± 937.5 of non-stimulated cells freely migrated to the bottom chamber. When Cyp A was present in this chamber, 58,589 ± 9161 cells were able to migrate. In these conditions, if the cells were previously activated with Con A, 104,418 ± 9342 cells were detected in the bottom chamber. This number was significantly decreased to control values, 40,000 cells (*p* < 0.001), when the cells were pretreated with CsA or Gracilin L, and to 63,768 ± 6367 cells (*p* < 0.01) after Gracilin A pretreatment before Con A stimulation. However, Gracilin H and Tetrahydroaplysulphurin-1 did not produce a significant reduction of T cells migration.

Next, we tested the effects of CsA and two *Spongionella* compounds *in vivo*, after a reaction of hypersensitivity in mice. The choice of compounds was done according to the results obtained in the migration assays, Gracilin H as representation of no effect over T cell migration assay and Gracilin L as representative of complete inhibition over this response. First, the pharmacokinetic behavior of Gracilin H and L as well as CsA after IP administration was checked in order to know if the compounds were absorbed through this administration route. In this way, mice were IP injected, and urine, feces, brain, and blood samples were analyzed. As Table 1 shows, after 1 h CsA treatment, the compound was detected in all the samples analyzed, although the highest amount was found in blood and urine samples. After 24-h treatment, the amount detected in blood and brain samples decreased, while in urine and feces, CsA was still high. In the case of Gracilins, both compounds were present in urine samples after 1- or 24-h treatment, while only Gracilin H was detected in feces. These results pointed to IP as a correct dosage route for these compounds.

**Figure 5:**
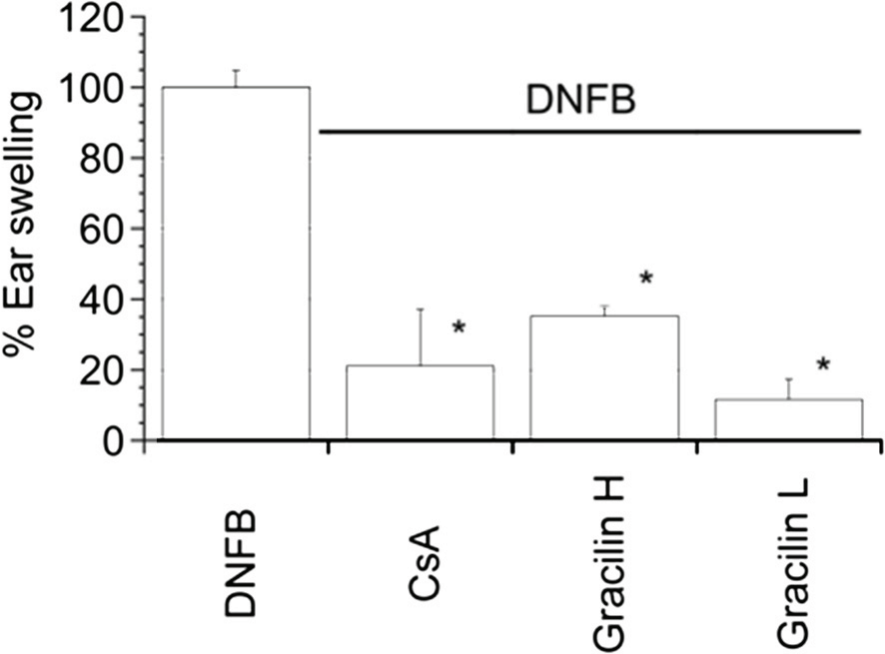
***In vivo* effect of CsA or *Spongionella* compounds on the ear thickness 24 h after DNFB challenge**. Mouse ear thickness calculated as the percentage of increment rate (thickness of the challenged ear minus the baseline thickness of the unchallenged ear) of ear swelling of DNFB-sensitized animals and compared to CsA (0.4 mg kg^−1^), Gracilin H (0.4 mg kg^−1^) or Gracilin L (0.4 mg kg^−1^) treated animals. *p < 0.05. Data are mean ± SEM of three mice per group.

Next, CsA and both Gracilins were tested on ear swelling after a DTH reaction. After 4–8 h from the last sensitization, DNFB-treated animals experimented marked edema and vasodilatation on the sensitized ear (data not shown). All animals were sacrificed 24 h after challenging. As shown in Figure 5, DNFB-treated animals were considered as the maximum% of ear swelling. Comparing CsA, Gracilin H-, or L-treated animals plus DNFB versus DNFB group, different responses on the % of ear swelling were observed. In the presence of CsA, ear swelling was 78.86 ± 16.07% (*p* < 0.05) reduced, similar to the effect observed with Gracilin L with a 88.37 ± 5.73% (*p* < 0.05) reduction, while the smaller effect was observed with Gracilin H that produced a reduction of a 64.75 ± 2.79% (*p* < 0.05).

**Figure 6:**
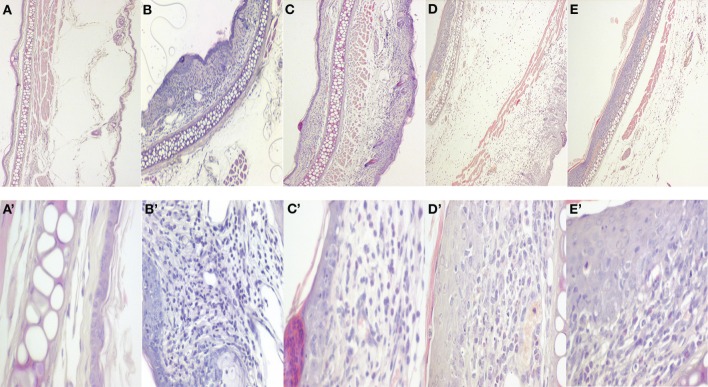
**Histological changes of CsA or *Spongionella* compounds treatment 24 h after DNFB–DTH-treated ear**. Ear tissues were stained with H-E and observed using a microscope. The observations were made at a magnification of ×4 for figures **(A–E)** and ×40 for figures **(A’,B,C,D’,E’)**. Microphotographs are representative from one animal. **(A,A’)**, non-challenged control group; **(B,B’)**, DNFB-sensitized group; **(C,C’)**, 0.04 mg kg^−1^ CsA-treated animals; **(D,D’)**, 0.04 mg kg^−1^ Gracilin H-treated group; **(E,E’)**, 0.04 mg kg^−1^ Gracilin L-treated group.

Furthermore, histological changes in ear tissues were also checked. Swelling of hypodermal tissue and the infiltration of inflammatory cells were observed in the ear section by histological analysis of H-E staining. As Figures 6B,B’ show, DNFB-treated cells significantly increased the ear thickness comparing to non-sensitized animal (Figures 6A,A’) as well as the number of inflammatory cells. Nevertheless, CsA-treated animals (Figures 6C,C’) mitigated DNFB-induced ear swelling, and inflammatory cells infiltration was observed. In a similar way, mouse treated with Gracilin H (Figures 6D,D’) and Gracilin L (Figures 6E,E’) underwent a reduction in ear inflammation as well as inflammatory cell infiltrates compared with DNFB-treated animals.

## Discussion

In previous results, we observed that the four *Spongionella-*isolated compounds bind to Cyp A with a similar binding affinity than CsA, mimicking the mechanism of action of this calcineurin inhibitor (22). Cyp A is a protein implicated in multiple processes, such as protein folding, trafficking, and transport (9). All these functions are related with the isomerase activity and are showed by all family members of Cyps (27). Thus, the expression of different type of membrane receptors, such as asialoglycoprotein receptor, insulin receptor, or receptor-type tyrosine–protein kinase, is regulated by this protein (28–31). The isomerase function of Cyp A is well documented for being inhibited by CsA (32). In this way, the transport and the expression of the surface membrane receptors aforementioned were greatly reduced in different reports by CsA treatment (25). Because *Spongionella* compounds directly targeted Cyp A (22), the reduction of the presence of the surface membrane receptor CD147 in T cells produced by all *Spongionella* compounds could be related with the modulation of the isomerase activity of the protein. Other reports proved the existence of a Cyp A binding site in the transmembrane region at the residue Pro^211^ of CD147 that is responsible for the receptor transport to the surface (9, 26, 33). In the case of *Spongionella* compounds, once they bind to Cyp A, they could modulate the trafficking of CD147 in a similar way than CsA. Moreover, soluble factors such as epidermal growth factor (EGF) or various inflammatory mediators such as interleukins have been shown to regulate CD147 transcription and translocation (11, 16, 18). Therefore, the inhibitory effect on EGF receptor and over IL-2 production by the four *Spongionella* compounds described in previous papers could be also under the reduction of the presence of CD147 in the cell surface (1, 22). On the other hand, it is well documented that Cyp A can be secreted to the extracellular medium as a response of the cell to stress (34, 35). In *in vitro* conditions, the release of Cyp A from the cytosol can be induced with lectins by activating the intracellular machinery of the T cells (34). Thus, we observed that once T cells were stimulated with Con A, they release Cyp A to extracellular medium. Nevertheless, with the exception of Tetrahydroaplysulphurin-1, Gracilins H, A, and L were able to efficiently reduce the secretion of Cyp A to extracellular environment. Reactive oxygen species (ROS) induce stress stimulus in the T cell and therefore can induce Cyp A secretion as described by many researches (34, 36). In accordance to previous results published with mouse cortical neurons, all *Spongionella* compounds were able to protect the cell against ROS insults by increasing nuclear factor E-related factor 2 levels in the nucleus, and consequently the antioxidant pathway that protects the cell is activated (19). Thus, such reduction of Cyp A secretion could be explained by the protective effects of these compounds stimulating different enzymatic cell defense mechanisms. Moreover, in *in vitro* and *in vivo* assays, all *Spongionella* compounds had the ability to inhibit extracellular signal regulated kinases (ERK), directly related with the inhibition of Cyp A secretion (19, 34). Besides, the implication of Cyp A in the CD147 transport to the plasma membrane, Gracilin A, and Gracilin L efficiently avoids the T cell migration in the presence of Cyp A. The effect of both compounds can be mainly attributable to the effect produced at intracellular level by reducing the expression of CD147 in the T cell surface and therefore decreasing the chemotactic effect for Cyp A when it is present in the extracellular medium. In the case of Gracilin L, it showed approximately two times less affinity than Gracilin H for the same target (22). Nevertheless, the full inhibition observed with Gracilin L over T cell migration comparing to the absence of effect of Gracilin H would not necessarily be associated with the affinity for the Cyp A, considering that we check the binding capacity with SPR on Cyp A, nevertheless, with this technique we cannot know the region where the compounds exactly bind to. Moreover, in *in vivo* experiments, both compounds disclosed a similar effect over ear swelling. This result can be explained by the effect of Gracilin H on other Cyps like Cyp B that is also involved in the inflammatory process (37). However, the effect of Gracilin H over other enzymes, transcription factors, or adaptor proteins, which induce a cascade of metabolic events result in the transcriptional activation of a large number of different pro-inflammatory genes (38). In fact, Gracilin H as well as Gracilin L reduced the expression of phosphorylated ERK in *in vivo* assays (20). In pharmacokinetics studies, we observed that CsA cross hematoencephalic barrier, in accordance with other researches, but it is detected 1 h after administration in a low concentration. However, in the case of Gracilin H and L, the absence of both compounds in blood could be explained by the association of the compounds to plasmatic proteins. With the IT–TOF technique, the masses range is *m/z* 50–5000, thus we could not detect the possible complex formed. Nevertheless, the presence in urine of Gracilin H as well as Gracilin L may point out a previous absorption and incorporation of the molecules to the bloodstream to reach the kidneys, where in order to being eliminated, they have to dissociate from the binded protein and are again in the equipe mass range. DTH reaction is usually regarded as a cell-mediated immune response and plays an essential role in the immune diseases. This is a pathologic response, type IV hypersensitivity reaction, involving T cell activation and the production of many cytokines (39). This type of hypersensitivity reaction is well-modulated by immunosuppressive drugs that possess a strong anti-DTH activity. In previous papers, we observed that *Spongionella* compounds mimic the effect of CsA on calcineurin and the translocation of NFAT to the nucleus. NFAT is one of the transcriptional factors that induce a cascade of metabolic events, resulting in the transcriptional activation of large number of different genes (38). In this way, they prevent the production of IL-2. These findings pointed out *Spongionella* compounds as possible anti-inflammatory or immunosuppressive agents (22). In the present study, we have shown that Gracilin H and Gracilin L efficiently reduced DTH reaction in the same way as CsA. Histological analysis of ear swelling showed a significant decrease on white cells infiltration when mice were pretreated with Gracilins or CsA. Thus, this effect can be produced by several mechanisms, such as the reduction of cytosolic calcium increase or the translocation inhibition of NFAT to the nucleus due to the calcineurin phosphatase activity reduction, both effects that lead to IL-2 inhibition (19, 21, 22). In addition, the reduction of CD147 expression in the cell surface produces the reduction of the inflammatory cells activation and proliferation and also by the reduction of Cyp A secretion due to the activation of antioxidant defense mechanisms of the cell avoiding the cell migration previously described (19, 20).

In this report, we show the ability of *Spongionella* compounds to reduce the cell membrane expression of CD147 receptor, the inhibition of Cyp A secretion, and the blockade of cell migration in the presence of Cyp A chemotactic stimulus. *In vivo* experiments confirmed the capacity of Gracilin H and Gracilin L to reduce mouse ear swelling, edema, as well as the early inflammatory cells, and among them T cells. The antioxidant and anti-inflammatory/immunosuppressive abilities described in previous works (19, 22) and the results obtained in the present research are good starting points for the promotion of Gracilins as valuable option for synthetic drug development in order to obtain new drugs and stem the problems that present some of the available drugs used with anti-inflammatory and immunosuppressive purpose.

## Author Contributions

All authors of this manuscript agree with the terms and conditions.

## Conflict of Interest Statement

The authors declare that the research was conducted in the absence of any commercial or financial relationships that could be construed as a potential conflict of interest.
